# Supplementary material for: A three-colour stress biosensor reveals multimodal response in single cells and spatiotemporal dynamics of biofilms
Source: NPJ Biofilms Microbiomes. 2023 Aug 21;9:57. doi: 10.1038/s41522-023-00424-1 (PMC10442448; doi:10.1038/s41522-023-00424-1)
Supplement: Supplementary file 2 — Reporting Summary [file 41522_2023_424_MOESM2_ESM.pdf]

nature portfolio

Corresponding author(s): Kersten S. Rabe

Last updated by author(s): Jul 16, 2023

Reporting Summary

Nature Portfolio wishes to improve the reproducibility of the work that we publish. This form provides structure for consistency and transparency in reporting. For further information on Nature Portfolio policies, see our Editorial Policies and the Editorial Policy Checklist.

Statistics

For all statistical analyses, confirm that the following items are present in the figure legend, table legend, main text, or Methods section.

n/a	Confirmed	
☐	☒	The exact sample size (n) for each experimental group/condition, given as a discrete number and unit of measurement
☐	☒	A statement on whether measurements were taken from distinct samples or whether the same sample was measured repeatedly
☐	☒	The statistical test(s) used AND whether they are one- or two-sided<br>*Only common tests should be described solely by name; describe more complex techniques in the Methods section.*
☐	☒	A description of all covariates tested
☐	☒	A description of any assumptions or corrections, such as tests of normality and adjustment for multiple comparisons
☐	☒	A full description of the statistical parameters including central tendency (e.g. means) or other basic estimates (e.g. regression coefficient) AND variation (e.g. standard deviation) or associated estimates of uncertainty (e.g. confidence intervals)
☐	☒	For null hypothesis testing, the test statistic (e.g. F, t, r) with confidence intervals, effect sizes, degrees of freedom and P value noted<br>*Give P values as exact values whenever suitable.*
☒	☐	For Bayesian analysis, information on the choice of priors and Markov chain Monte Carlo settings
☒	☐	For hierarchical and complex designs, identification of the appropriate level for tests and full reporting of outcomes
☒	☐	Estimates of effect sizes (e.g. Cohen's d, Pearson's r), indicating how they were calculated

Our web collection on statistics for biologists contains articles on many of the points above.

Software and code

Policy information about availability of computer code

Data collection — *Provide a description of all commercial, open source and custom code used to collect the data in this study, specifying the version used OR state that no software was used.*

Data analysis	Data analysis described in Methods, section "RNA-seq data processing and analysis": The sequence reads were quality checked using FastQC v0.11.9 (www.bioinformatics.babraham.ac.uk/projects/fastqc) and quality-trimmed using Trim Galore. The rRNA reads were removed using SortMeRNA v4.3.4 (silva-bac-16s-id90 and silva-bac-23s-id98 databases for reference). The filtered reads were mapped to the E. coli MG1655 (ASM584v2) genome, edited to include the sensor construct sequences, with the STAR aligner v2.7.6a. Read counts for each gene were determined using the featureCounts. Counts were normalized and differentially transcribed genes were analysed (padj = < 0.05) with DESeq2 in R v3.6.3. The design for DESeq2 was set to test for variation between the four different RGB-S E. coli subpopulations. .

For manuscripts utilizing custom algorithms or software that are central to the research but not yet described in published literature, software must be made available to editors and reviewers. We strongly encourage code deposition in a community repository (e.g. GitHub). See the Nature Portfolio guidelines for submitting code & software for further information.

Data

Policy information about availability of data

All manuscripts must include a data availability statement. This statement should provide the following information, where applicable:

- Accession codes, unique identifiers, or web links for publicly available datasets
- A description of any restrictions on data availability
- For clinical datasets or third party data, please ensure that the statement adheres to our policy

All genetic information used or generated throughout this study were made available in the Supplementary Table 2. RNA-seq data was submitted to the National Centre for Biotechnology Information's (NCBI) Gene Expression Omnibus (GEO) under accession number GSE211360. The materials and data that support the findings of this study are available from the corresponding author upon reasonable request.

Research involving human participants, their data, or biological material

Policy information about studies with human participants or human data. See also policy information about sex, gender (identity/presentation), and sexual orientation and race, ethnicity and racism.

Reporting on sex and gender	not applicable
Reporting on race, ethnicity, or other socially relevant groupings	not applicable
Population characteristics	not applicable
Recruitment	not applicable
Ethics oversight	not applicable

Note that full information on the approval of the study protocol must also be provided in the manuscript.

Field-specific reporting

Please select the one below that is the best fit for your research. If you are not sure, read the appropriate sections before making your selection.

☒ Life sciences ☐ Behavioural & social sciences ☐ Ecological, evolutionary & environmental sciences

For a reference copy of the document with all sections, see nature.com/documents/nr-reporting-summary-flat.pdf

Life sciences study design

All studies must disclose on these points even when the disclosure is negative.

Sample size	six (unless otherwise indicated)
Data exclusions	not applicable

Replication	all experiments were conducted in biological replicates
Randomization	Populations of bulk, random cells were used for biosensing
Blinding	Cell populations that were treated in independent replicates with different stressors were labelled with the names of their chemical stressors and their data were analyzed accordingly

Reporting for specific materials, systems and methods

We require information from authors about some types of materials, experimental systems and methods used in many studies. Here, indicate whether each material, system or method listed is relevant to your study. If you are not sure if a list item applies to your research, read the appropriate section before selecting a response.

Materials & experimental systems

n/a	Involved in the study
☒	☐ Antibodies
☒	☐ Eukaryotic cell lines
☒	☐ Palaeontology and archaeology
☒	☐ Animals and other organisms
☒	☐ Clinical data
☒	☐ Dual use research of concern
☒	☐ Plants

Methods

n/a	Involved in the study
☒	☐ ChIP-seq
☐	☒ Flow cytometry
☒	☐ MRI-based neuroimaging

Flow Cytometry

Plots

Confirm that:

☒ The axis labels state the marker and fluorochrome used (e.g. CD4-FITC).

☒ The axis scales are clearly visible. Include numbers along axes only for bottom left plot of group (a 'group' is an analysis of identical markers).

☒ All plots are contour plots with outliers or pseudocolor plots.

☒ A numerical value for number of cells or percentage (with statistics) is provided.

Methodology

Sample preparation	Culture preparation and cell sorting. The RGB-S E. coli strain was treated with 2-propanol 2% (v/v) for 5h at 37 °C in two cultures of 500 ml each, utilizing the standard stress assay protocol described above. Untreated cultures were prepared under the same conditions as the control. Control cells were then diluted with 1X phosphate buffered saline (PBS) to an approximate concentration of 1 x 106 cells ml-1 and analyzed in the cell sorter to set the background fluorescence signal threshold for the sorting of the treated samples. Afterwards, populations from RGB-S E. coli treated cells were also diluted with PBS and sorted using a MoFlo XDP High-Speed Cell Sorter (Beckman Coulter) equipped with 405 nm, 488 nm, and 561 nm laser lines. Post-acquisition analysis was done with FlowJo software (BD Biosciences).
Instrument	MoFlo XDP High-Speed Cell Sorter (Beckman Coulter)
Software	FlowJo software (BD Biosciences)
Cell population abundance	One millions cells were sorted for each sample (except for RFP-2 and RFP-GFP-2 which received 0.7 and 0.75 million cells, respectively) into 250 µl of RNAPure™ peqGOLD (VWR International GmbH), then immediately vortexed and kept on ice until extraction.
Gating strategy	Both non-stressed control sample and subpopulations of stressed mono RFP, mono GFP, dual RFP-GFP, triple RFP-GFP-BFP with stress fluorescence signals higher than the control background threshold were sorted in duplicates. Gating of the aforementioned populations is shown in Supplementary Figure 10.

☒ Tick this box to confirm that a figure exemplifying the gating strategy is provided in the Supplementary Information.
